# Draft Genome Sequence of a Violacein-Producing *Iodobacter* sp. from the Hudson Valley Watershed

**DOI:** 10.1128/genomeA.01428-17

**Published:** 2018-01-04

**Authors:** Georgia Doing, Gabriel G. Perron, Brooke A. Jude

**Affiliations:** a Department of Microbiology and Immunology, Geisel School of Medicine at Dartmouth, Hanover, New Hampshire, USA; b Reem-Kayden Center for Science and Computation, Program of Biology, Bard College, Annandale-on-Hudson, New York, USA

## Abstract

*Iodobacter* species are among a number of freshwater Gram-negative violacein-producing bacteria. *Janthinobacterium lividum* and *Chromobacterium violaceum* have had their whole genomes sequenced and annotated. This is the first report of a draft whole-genome sequence of a violacein-producing *Iodobacter* strain that was isolated from the Hudson Valley watershed.

## GENOME ANNOUNCEMENT

*Iodobacter* species have been observed in freshwater samples, often favoring psychrophilic conditions, such as glaciers and Arctic lakes (1). *Iodobacter* spp. have also been reported in pathogenic lesions on freshwater trout species (2). *Iodobacter* spp. are Gram-negative motile bacilli. They are often violet pigmented, resulting from the expression of a five-gene *vio* operon to produce violacein (3). This compound was recently linked to a killing effect on an amphibian-specific fungus, *Batrachochytrium dendrobatidis* (1, 4–6).

BJB302 was isolated when Hudson Valley freshwater sources were inoculated on R2A agar and incubated at 22 to 25°C for 48 h. This strain presented as deep-violet-pigmented colonies on LB and 1% tryptone agar media. This strain has been examined for growth phenotypes and behaviors, as well as surface motility properties (B.A.J., unpublished data).

Genomic DNA was extracted using the Qiagen Gentra Puregene Yeast/Bact. kit, according to standard protocols. Paired-end Illumina libraries (150 bp) were prepared, and Illumina HiSeq 4000 sequencing was performed by Wright Labs (Huntington, PA). Reads were assembled utilizing a modified version of a previously published local pipeline (7). Briefly, adapters and contaminants were removed, and reads were quality filtered using BBDuk from the BBMap package version 37.50, using a *Q* score cutoff of 10 (https://sourceforge.net/projects/bbmap). The draft whole-genome assembly was constructed with SPAdes version 3.11.0 (8), using the following k-mer sizes: 21, 33, 55, 77, 99, and 127. Contigs shorter than 500 bp or contigs that were composed of fewer than four reads were subsequently filtered out of the assembly. Assembly was examined for optimization using the SSPACE and GapFiller programs (9–11). The draft whole-genomic assembly resulted in 204 contigs, with an *N*_50_ of 54,392 bp and median coverage of 272-fold. The approximate genome size is predicted to be 4.8 Mbp in length, with a G+C content of 49.59%.

The assembled contigs were annotated using several methods, including a local pipeline running the Prokka genome annotation software (12, 13), the RASTtk annotation software, via the PATRIC pipeline (14, 15), and the NCBI Prokaryotic Genome Annotation Pipeline (PGAP) (16). The 16S rRNA BLAST results were 99% identical to *Janthinobacterium* B9-8, as well as *Iodobacter arcticus* and other *Iodobacter* species. Annotations for BJB302 yielded an average of 4,550 coding sequences (CDSs). As expected, the violacein biosynthesis operon (*vioA–E*) was present in all strains. Additionally, genes associated with chemotaxis-mediated biofilm dispersion (*bdlA*), twitching motility (*pilT, pilJ, pilH,* and* pilG*), and quorum sensing (*luxQ*) were noted.

Due to the vibrancy of the violacein pigment production in this strain and the apparent lack of biofilm production (data not shown), work is currently being conducted to elucidate the mechanism of violacein regulation and production within the aquatic environment.

### Accession number(s).

This whole-genome shotgun project has been deposited at DDBJ/ENA/GenBank under the accession number PDZG00000000. The version described in this paper is version PDZG01000000.
